# Reminiscences of Half a Century Ago

**Published:** 1910-08-27

**Authors:** Carey Coombs


### SPECIAL ARTICLE.

### REMINISCENCES OF HALF A CENTURY AGO.

By CAREY COOMBS, M.D. Lond.

Strictly speaking my training in medicine began more than fifty years ago, when a relation undertook to teach me the first principles of the art. Happily he was a man of rare powers, who practised, as did so many men in those days, on their observation and experience, and who read nothing medical. And yet he and his like succeeded in curing patients and obtained the love and respect of thousands with whom they had to do.

### Diagnostic Instruments.

All those years ago, some of them used microscopes, always English made; German microscopes were despised even more than those which came from France. A "Ross" with a Powell and Lealand objective formed the climax of the instruments; but not always to look at blood, or sputum, or casts. Many men found relaxation in making out the markings on diatoms—*e.g.* Coccinodiscus radiatus and the varieties of Pleurosigma were great favourites. Was there any charm about their names? Probably the same charm which seems to attach to the long-named bacilli and cocci and spirilla. Surely Staphyloccocus pyogenes aureus, like an old-fashioned German Count, owes much of his prestige to his long name. Others beside the old woman of history find comfort in that precious word "Mesopotamia." In the sixties the organic chemists held the best place as regards long names, conscientiously putting the composition of a complex thing into the name, bit by bit. A great impulse to the use of first-class objectives was given when bacilli were first sought in stained sputum about forty years ago. Then it was that the very steady and yet portable stands made in Germany began to be generally used in England. Systematic blood counting and hæmoglobin measurements were unknown in the early sixties. The use of the dried blood film is still more modern.

The laryngoscope and ophthalmoscope began to be used in my student days. Dr. Johnson, of King's, with the first instrument showed us his vocal cords in a looking-glass in a ward in St. Mary's Hospital. Mr. Ernest Hart used a demonstrating ophthalmoscope to give his pupils their first sight of a fundus about 1862.

### Electricity.

High-tension electricity has come into use in the Röntgen rays, and incandescent lamps have revealed much that is interesting in the nasal fossæ, the stomach, and bladder. In this half-century the thermometer has been introduced; my first instrument, 10 inches long, was carried in a wooden case. But it soon was reduced to 6 inches, and then made self-registering. A middle-aged tradesman suffering from laryngitis solemnly told me that he would rather die than have "that thing" put in his armpit. The binaural stethoscope was first seen by me in 1870, in Dr. Sibson's consulting-room in Brook Street. It was a corner house in which he lived, and the busy traffic would have drowned lung sounds as heard through the old Laennec instrument. I think the late Sir William Broadbent used that same stethoscope after Dr. Sibson's death, almost until the end of his practice. The addition of a microphone to the binaural is a great gain.

Fifty years ago the use of the galvanic current was almost unknown, and even now few men realise its value enough to use the current from the public supply or their own portable batteries. Fifty years ago the Listerian methods were used by men, only unconsciously; for instance, Friar's balsam on strips of lint foreshadowed Sir Joseph's protective dressing. An amusing instance of this instinctive use of good remedies occurred in this neighbourhood almost eighty years ago. The medical man in attendance on a boy suffering from typhoid advised the mother to take the patient into the garden, place him in a hole in the earth, and cover him over with the soil, leaving him in his earth bath for half an hour. The boy's condition was so much improved that the earth bath was used again and again. Some Continental surgeons have used dry earth as a dressing for wounds. The possibility of keeping an open wound (whether traumatic or operative) antiseptic had not been believed in the sixties; but since that discovery surgery is a different art. An American cyclist did not then find it necessary to keep a notice in the tool-bag to inform any surgeon finding him unconscious that his appendix had already been removed—twice.

### The Genesis of Asepsis.

At the same time those who have watched the progress of antiseptic treatment have seen many changes in the "infallible" methods, and such inconsistencies as that of a good surgeon who was so particular about the accurate dilution of his carbolic lotion that while he was doing it by means of a small bottle, the daylight declined. As there was no electric lighting in that place, a difficult dissection had to be finished in twilight. Another case of antiseptic caution occurred when I was anæsthetising a lady for ovariotomy done by the pioneer of that operation. He had a large spray-producer boiling in readiness to clear the air of "germs," but, alas! he forgot to turn the spray on until the sutures had been put in. Yet the patient survived.

It was at the Obstetrical Society, I believe, that this operation was condemned as unjustifiable, just fifty years ago; yet now the peritoneum receives about as much respect as the skin. Forty years have passed since my first amputation, but it is pleasant to know that though the methods have been much improved since then, yet the arteries were twisted, the wound was carefully cleaned with weak carbolic lotion, and the dressings were strictly Listerian. The wound was not opened for twenty-one days, and had then united throughout, only two-thirds of an inch of skin wound being unhealed.

At the first large operation I watched a full dose of brandy was the only anæsthetic given. Chloroform was usually given for my own operations (diluted with ether and alcohol) on a pocket handkerchief, which method seems to have survived the uncomfortable large towel which concealed the face and chest. The great gas-bag which the anæsthetist bore on his back, looking like Christian in the "Pilgrim's Progress," and even the clever Junker air-and-chloroform mixing apparatus have both nearly disappeared. Probably these things gave a false sense of safety, and the patient is now more carefully watched.

### Operations Then and Now.

Operations on fractures were almost unknown fifty years ago; nothing of that kind has been really called for in my practice, though two or three of the patellar fractures would probably have done better if wired. In twenty or thirty cases excellent union has been obtained by raising the leg and applying pressure above and below the fracture; in one case needles were passed above and below the bone and tied together with strong ligatures. The result was excellent.

Excision of the patellar bursa is not a real advance, seeing that much simpler methods will remove the trouble. Perhaps the attention of the public has been called to this disorder by Jerome's allusions in "Three Men in a Boat." In the last half-century fixation of bones and joints in the treatment of fractures and diseases has wonderfully improved. Sayre's plaster-of-Paris splint for spinal disease is now applied, I believe, without the hanging to which we used to condemn our patients.

### Heat and Hydro-Therapeutics.

Heat in the form of the actual cautery was being used less and less, but revived in the gas cautery, then in the Paquelin thermo-cautery (for which the instrument used in "poker work" is a good substitute), and, best of all, in galvanocautery.

The regular use of the air bath and of the hot chamber for single rheumatic joints, heated by the use of electricity, and the wonderful irradiation methods by the Finsen lamp and radium have all been established as valuable remedies in this period. Within my memory hypodermic medication, including the use of antitoxins and vaccines, and cocaine local anæsthesia have been adopted. Nitrous oxide and ethyl chloride were not used until the middle or end of the century.

Until the last half of the century the man with a dilated stomach did not have a stomach tube passed; let us now congratulate him if by that means he escapes an operation. Let us also congratulate the possessor of a large prostate that he can have it removed, or, if he likes to keep it, that his bladder can be kept clean, and that cystitis is not the rule in such cases, as it was fifty years ago. To pass an unsterilised catheter now is an assault on a man or woman.

### The Encroaches of the Surgeon.

The diabetic patient is now no longer safe from the surgeon. If certain crystals can be found, showing that his pancreas is unsound, surgeons are ready to explore that organ. The much extended sphere of surgery has tended to make the younger men think rather lightly of the value of medicine. Sir Dyce Duckworth has said in his article on "The Medical Student and his Work" that men do not know how to use opium and alcohol; but, happily, there are many men in practice who are not afraid of a cant phrase—I am thinking of "masking the symptoms," which, in plain English, is "you must put up with your pain until I am ready to remove your appendix." With regard to alcohol, it is probable that many tinctures owed their efficiency to the presence of the stimulant.

There are certain valuable remedies which have been in use for so many years that one cannot fix their age. But salicine and the salicylates, strophanthus, phenacetin, antipyrin, urotropin, the various forms of cocaine, chloral, some forms of bromine, all these have come into use within fifty years. Carbolic acid was beginning to be used; bichloride of mercury was not then used as an antiseptic.

In 1860 most patent foods, the concentrated animal juices, such as Liebig's Extract and Benger's, and nearly all the infant foods, were unknown. Happily, people had not then so largely adopted the custom of tippling mineral waters nor acquired the taste for taking sulphate of soda out of bottles filled at foreign spas, after the simple drug had been handed over to horses and other beasts.

### Hospitals and Hospital Staffs.

Hospital conditions have changed much in fifty years. Physicians no longer wear their hats in the wards, inflated house surgeons are rather the better for being controlled by the resident medical officer; but really the house surgeon demands a whole chapter. He is not as independent now as we were, but, on the other hand, he is allowed to do many more operations than were permissible in my time, when we were limited to tracheotomy. At St. Mary's this change was largely due, I believe, to Mr. Owen. It is unquestionable that the medical student to-day is a much better man than fifty years ago. Albert Smith and Charles Dickens would scarcely write such caricatures in these days as they wrote then. The frequent examinations are much better than the short interview at the College of Surgeons which decided a man's fitness for practice. About sixty or seventy years ago that matter was settled in about forty minutes, after the student had finished his hospital course of two winters and one summer, really a year and a half. His general education was taken for granted before 1860, or thereabout. He has his clinical teaching now by the bedside, and has generally more practical instruction. There are still too many lectures; practical work in the hospital, which is the privilege of those holding appointments, makes the good practitioner, general or special. The University of London, by maintaining a high standard in the matriculation examination, weeded out those young men whose education had been neglected, or who had not the power to learn, and subsequently insisted that each man before taking his degree should have had six months of special care of patients.

Nurses.

But the greatest change in hospital life is in the rank, training, and efficiency of nurses. Their ranks are recruited from a class of women quite different from the class supplying them in my student days; we actually had some nurses imported directly from the Lock Hospital, where they had qualified not as nurses but as patients. One change in hospitals is to their great discredit—the increase of the out-patients. In London and large towns they exceed one-third of the whole population, having increased immensely in the past half-century. Surely it is a terrible wrong to a medical man, whose father has spent £800 or £1,000 in qualifying him for practice, to provide medicine and advice with no charge at all for the very people who should support him when he commenced practising. No wonder that the practitioners in South London united in appealing to the Governors of King's College Hospital not to remove that Institution into their neighbourhood.

In the sixties almost all general practitioners supplied medicine, and probably half of those who live in large towns continue to do so still.

Drugs.

A great change has come over the administration of drugs; their compression into tabloids is an improvement in some ways. The same may be said of telephones and motor-cars as was said to me by an old lawyer fifty years ago with regard to railways and telegraphs, "When I was young, if a man in London wanted papers sent to him, he gave you a fortnight before you sent them by the coach; now you have a wire in the morning asking you to send your papers in four or five hours." As an elderly man he felt the pressure of modern life. Though the medical man must feel the increased strain which the motor-car brings, he gains immensely in the matter of time.

Midwifery Practice.

The practice of midwifery has much improved; the businesslike way in which a clever man extracts a baby with the help of a little chloroform takes away so much suffering, especially in first labours. In course of time, if it became general, this dexterous treatment might lead to increase in the population. Fifty years ago the poor young primipara had two or three days of suffering, which was then considered quite the proper thing by everyone but herself. Many such cases are now shortened to a fourth of their former duration. There is much less pain than in the old days. A nurse at a large hospital said to me, "We never let a patient suffer long from any cause; we go to the sister to fill the hypodermic syringe." Outside the hospital there is the same feeling; everyone has heard of brutal, unfeeling doctors; but who sees them in the present day?

It is fitting that the members of the noblest profession should themselves become nobler with each year of their career.

